# Global evolution of breast cancer incidence in childbearing-age women aged 15–49 years: a 30-year analysis

**DOI:** 10.1007/s00432-025-06113-0

**Published:** 2025-02-11

**Authors:** Chengwei Xia, Yini Liu, Wei Yong, Xin Qing

**Affiliations:** 1 Department of Thyroid & Breast Surgery, Chengdu Seventh People’s Hospital (Affiliated Cancer Hospital of Chengdu Medical College), Chengdu, China; 2 West China Hospital, Sichuan University, Chengdu, China

**Keywords:** Global burden of disease study, Women of child-bearing age, Breast cancer, Age-period-cohort, Epidemiology

## Abstract

**Background:**

Breast cancer (BC) poses an increasing threat to women’s health, yet its characteristics in women of childbearing age (WCBA) are infrequently reported. This study aims to investigate the patterns and trends in BC incidence among WCBA over the past decades.

**Materials and methods:**

This study focuses on BC incidence in women aged 15–49 years, consistent with the WHO definition of WCBA. Estimates and 95% uncertainty intervals (UIs) for BC incidence in WCBA were obtained from the Global Burden of Diseases Study 2021. We utilized an age-period-cohort (APC) model to estimate the overall annual percentage change in incidence (net drift, % per year) and the annual percentage change within each age group (local drift, % per year). This model also provided fitted longitudinal age-specific rates adjusted for period deviations (age effects) and period/cohort relative risks (period/cohort effects) from 1992 to 2021.

**Results:**

In 2021, the global incidence of BC among WCBA was 561.44 thousand (95% UI 519.76 to 606.99). Between 1992 and 2021, the estimated annual change in BC incidence among WCBA was 0.47 (95% CI 0.41–0.52) worldwide, ranging from −0.43 (95% CI −0.54–−0.31) in High sociodemographic index (SDI) region to 2.03 (95% CI 1.97–2.1) in Low-middle SDI region. Local drift analysis showed that higher SDI regions had higher age-standardized incidence rates among WCBA, with age effects demonstrating similar patterns across different SDI regions and increasing risk with age. Notably, the rising trend in BC incidence among WCBA occurs at progressively younger ages. Globally, unfavorable period and cohort effects were observed. All SDI regions exhibited increased period and cohort risks, except for the High SDI region, which saw a reduction in incidence rates influenced by period and cohort effects, particularly among those born after 1996.

**Conclusion:**

The increasing incidence of BC among WCBA highlights the urgent need for effective intervention and preventive policies to alleviate this growing global burden.

**Supplementary Information:**

The online version contains supplementary material available at 10.1007/s00432-025-06113-0.

## Introduction

Breast cancer (BC) is the leading malignancy among women worldwide, contributing significantly to morbidity and mortality (Siegel et al. 2024; Sung et al. 2021). While traditionally incidence rates climb with age, a notable trend in recent decades is the increasing prevalence of BC among women of childbearing age (WCBA), typically aged 15–49 (Sun et al. 2021; Shah et al. 2019). The risk of BC slightly escalates as girls progress through adolescence, yet it remains relatively infrequent in this demographic (Terry and Colditz 2023; Burstein et al. 2021). Concurrently, recent data indicate that BC is the predominant cancer among young adults aged 15–39, with approximately 10% of all BC cases in the United States occurring in women under 45 (Giaquinto et al. 2022).

Accumulative evidence indicates that women under 50 are more prone to exhibit aggressive BC subtypes and advanced stages, as well as a higher likelihood of enduring therapy-related side effects and psychosocial challenges post-diagnosis (Liu et al. 2020; Loibl et al. 2024). Notably, routine screening for BC is not generally advised for younger women, which poses challenges for early detection. The incidence of metastatic BC in women aged 25–39 has risen annually by 2.1% between 1976 and 2009 (Johnson et al. 2013). Adolescent and young women consistently exhibit lower BC survival rates than their older counterparts (Johnson et al. 2018). Significantly, an international study underscores a link between lower socioeconomic status and a higher likelihood of late-stage BC diagnosis (Huang et al. 2021; Xu et al. 2023). Additionally, the risk of chemotherapy-induced premature ovarian failure is considerable, adding to the unique challenges of fertility preservation and pregnancy management faced by WCBA with BC (Moore et al. 2015).

This demographic confronts distinct physiological and social challenges, encompassing reproductive health, career progression, and family obligations, thereby amplifying the profound implications of a BC diagnosis (Maffoni et al. 2017). Hence, evaluating the burden of BC among WCBA is imperative for launching targeted intervention initiatives on a global scale. This research utilizes comprehensive epidemiological data from the Global Burden of Disease Study (GBD) 2021 to explore the temporal and geographical trends in BC incidence among WCBA across the past three decades.

## Methods

### Data extraction

This study's BC data for WCBA is sourced from the GBD 2021, offering the most recent epidemiological estimates on the burden of 371 diseases and injuries across 21 GBD regions and 204 countries and territories, spanning several decades. BC cases were identified using ICD-10 codes (C50-C50.629, C50.8-C50.929, Z12.3-Z12.39, Z80.3, Z85.3, Z86000), including invasive diagnosis, pre-invasive diagnosis and screening. Data were drawn from hospital records, population-based registries, and community surveys to ensure broad coverage, with robust statistical modeling addressing inconsistencies. The GBD framework uses rigorous statistical modelling to reconcile data from different sources. Techniques used include age standardization and adjustments for missing or incomplete data to ensure consistency and comparability across regions and time. The methodologies utilized in the GBD database have been extensively detailed in prior literature (GBD 2021a, 2021b, 2021c). GBD 2021 metrics encompass estimates alongside their 95% uncertainty intervals (UIs), determined by the 25th and 975th percentiles of the ordered 1000 estimates, as per the GBD algorithm. Furthermore, GBD 2021 introduced a sociodemographic index (SDI) for each country and territory, as an integrative measure of economic income, educational attainment, and fertility levels (GBD 2019). Utilizing GBD 2021 SDI values, countries were stratified into one of five quintiles: Low, Low-middle, Middle, High-middle, and High. This study adheres to the WHO's definition of WCBA as women aged 15–49 years (Women’s health. 2024). The study further delineates seven age brackets to discern temporal trends in the age-specific incidence rates: 15–19, 20–24, 25–29, 30–34, 35–39, 40–44, and 45–49 years.

### Analysis of overall temporal trends in BC incidence

This study evaluates the overall temporal trends in BC incidence among WCBA by examining both the raw numbers and the age-standardized rates from 1992 to 2021. The age-standardized incidence rate of BC in WCBA was estimated using the direct method of age standardization, an approach that treats rates as a weighted sum of independent Poisson random variables (Butler and Kalasinski 1989; Fay and Feuer 1997). Additionally, the estimated annual percentage change (EAPC), a widely recognized and effective measure, has been extensively utilized in prior research to monitor trends in indicators across defined time frames (Liu et al. 2019). The EAPC is calculated by fitting the natural logarithm of the incidence rates into a regression model with time as the independent variable, projecting the natural logarithm of each observation onto a linear regression, and deriving the EAPC from the slope of this line.

### Age-period-cohort modeling analysis of incidence data

This study conducted an age-period-cohort (APC) modeling analysis on BC incidence data among WCBA to investigate the complex interrelationships between age, period, and cohort effects on observed trends (Bell 2020). The APC model serves as a versatile analytical instrument, decomposing incidence rates across age, period, and cohort dimensions, thereby offering an in-depth examination of temporal dynamics (Rosenberg et al. 2014; Rosenberg and Anderson 2011). Parameters of the model were estimated utilizing statistical software, with identifiability concerns mitigated by the software’s built-in algorithms, ensuring a precise delineation of the effects. Model performance was evaluated through the Akaike Information Criterion (AIC) and Bayesian Information Criterion (BIC) to validate the robustness of the results. The analyses uncovered significant trends, and the annual percentage changes in specific rates shed light on the evolution of BC incidence over the study period.

In preparing the APC model’s input data, we utilized incidence estimates for BC in WCBA and population data for each country and region, as reported in the GBD 2021. To ensure consistency, age intervals should match period intervals; hence, 5-year age groups were paired with 5-year calendar periods. Accordingly, the study's entire timeframe (1992–2021) was segmented into six 5-year intervals: 1992–1996, 1997–2001, 2002–2006, 2007–2011, 2012–2016, and 2017–2021. Consequently, the analysis incorporated 12 partially overlapping 10-year birth cohorts, specifically: 1942–1951, 1947–1956, 1952–1961, 1957–1966, 1962–1971, 1967–1976, 1972–1981, 1977–1986, 1982–1991, 1987–1996, 1992–2001, and 1997–2006. The categorization of subjects into 12 overlapping 10-year birth cohorts serves several purposes. First, it allows for a more detailed examination of cohort effects by ensuring smooth transitions and continuity between successive groups. Overlapping cohorts reduce potential information gaps and capture finer variations in temporal trends. Second, this structure enhances the precision of APC analysis by balancing statistical robustness with temporal resolution. Third, overlapping cohorts facilitate the identification of long-term trends in BC incidence across successive generations, accounting for changes in environmental exposures, lifestyle factors, and healthcare advancements over time.

The APC models were utilized to evaluate both overall and age-specific trends in disease incidence (Rosenberg et al. 2014). The net drift within these models captures the annual percentage change in overall incidence by accounting for both temporal and cohort effects, whereas local drift offers insights into variations within specific age groups. While the annual drift may appear minimal, its cumulative impact over several decades can be substantial. The statistical significance of these trends is determined by the APC model through Wald's chi-square test. This test delineates the age effect through longitudinal ratios that are adjusted for period variations, and it measures the period or cohort effect by comparing the relative risk to a reference value. The selection of a reference year is inconsequential for interpreting the results, as the model's comparative nature remains consistent. Employing R software (version 4.3.3), the analysis and visualization of the model were conducted meticulously, ensuring a rigorous examination of incidence trends. Statistical significance was denoted by p-values below the threshold of 0.05.

### Ethics and STROCSS statemen CC

Ethical approval is not required for the use of anonymized, publicly available epidemiological data, and patient informed consent is not required to access and download data from the database. This work is reported in line with the strengthening the reporting of STROCSS criteria (Supplemental Digital Content) (Mathew et al. 2021).

## Results

### Trends in BC incidence in WCBA, 1992–2021

**Fig. 1:**
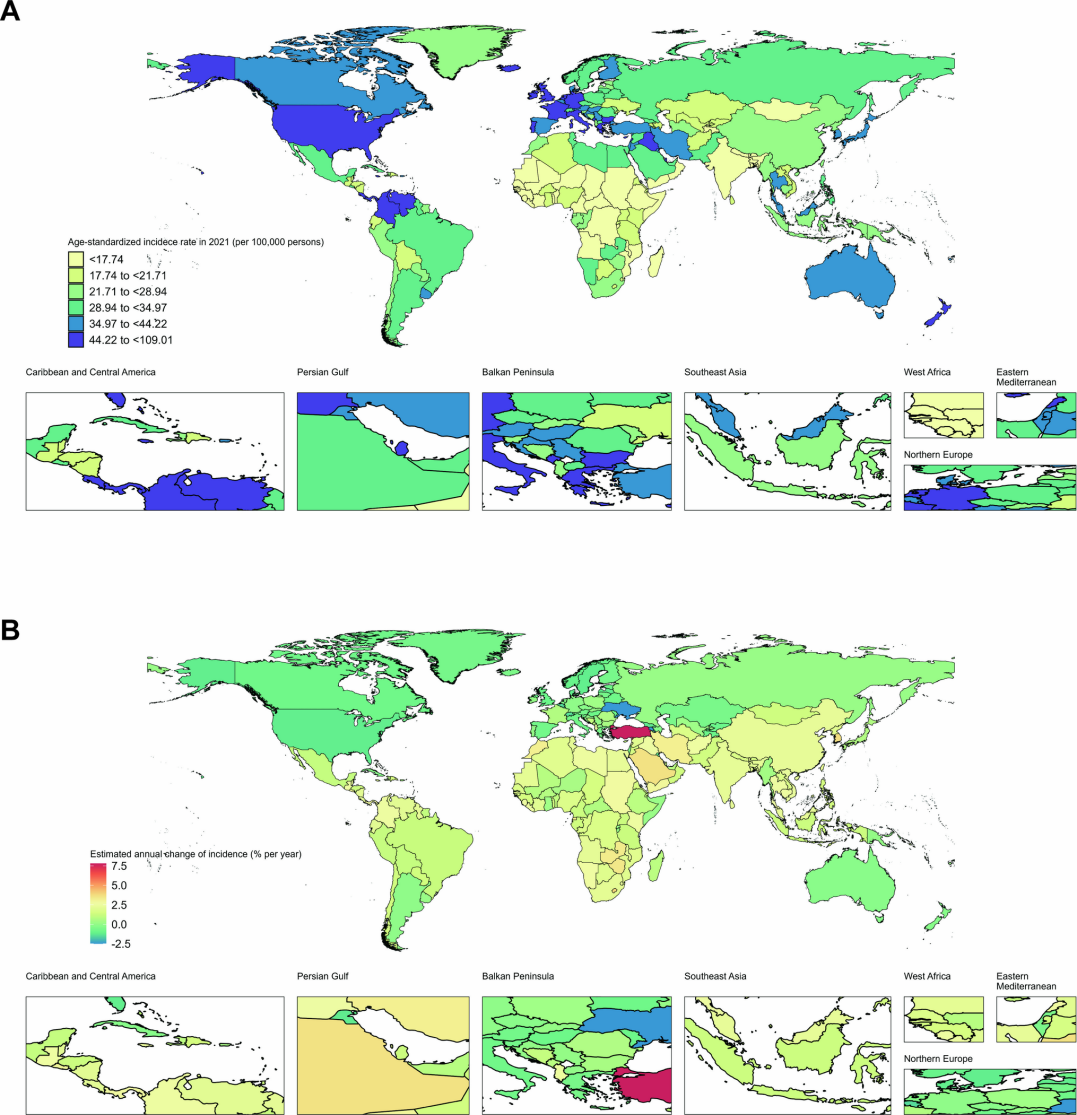
Age-standardized incidence rates of BC among WCBA in 2021 (**A**), alongside the estimated annual percentage change in incidence from 1992 to 2021 (**B**) across 204 countries and territories. *BC* breast cancer; *WCBA* women of childbearing age

Table 1 presents global and regional female populations, incident numbers, age-standardized incidence rates, and net drift of incidence. Between 1992 and 2021, the global population growth corresponded with a roughly 102.31% increase in new BC cases among WCBA, totaling 561.44 thousand in 2021 (95% UI 519.76–606.99). The incident number experienced a percentage increase across all SDI quintiles. In 2021, the global age-standardized incidence rate of BC for WCBA was 27.51 per 100,000 persons (95% UI 25.46–29.75), marking a 0.47% annual rise since 1992. A relative decrease in the age-standardized incidence rate was observed in the High SDI quintile. Moreover, the APC model estimated the global net drift of BC incidence among WCBA to be 1.15 per year (95% CI 1.08–1.21), with the range extending from 0.02 per year in the High SDI quintile (95% CI −0.09–0.14) to 2.33 per year in the Low-middle SDI quintile (95% CI 2.24–2.42). Figure 1 and Table S1 depict the national incident numbers, the age-standardized incidence rates for 2021, and the estimated annual changes in incidence from 1992 to 2021 for BC among WCBA. In 2021, among the 204 countries and territories, 10 reported at least 10,000 incident cases, with China, India, the USA, Brazil, and Indonesia accounting for the majority at 44.63% of global BC incidence among WCBA. Meanwhile, Monaco had the highest age-standardized incidence rate at 109.01 per 100,000, followed by Panama at 67.88 per 100,000 and the Cook Islands at 62.51 per 100,000, all of which were more than double the global average. Between 1992 and 2021, Turkey recorded the highest net drift and EAPC in incidence rates, at 8.73% (95% CI 7.97–9.49%) and 7.8% per year (95% CI 6.72–8.89%), respectively. In 2021, the age-standardized incidence rate in Turkey was 40.16 per 100,000 persons (95% CI 26.82–56.59). The APC model's net drift estimates indicated that the majority of the 204 countries and territories showed an increasing trend in BC incidence, highlighting significant heterogeneity in BC patterns worldwide.

**Table 1:** Breast cancer incidence trends in WCBA across Socio-demographic Index quintiles, 1992–2021

	Global		High SDI		High-middle SDI		Middle SDI		Low-middle SDI		Low SDI	
	1992	2021	1992	2021	1992	2021	1992	2021	1992	2021	1992	2021
Female population												
Number (x1,000,000)	2727.07	3931.96	452.82	548.36	545.38	651.47	873.06	1215.35	591.96	955.39	261.25	558.27
Incidence												
Number (x1,000)	277.81 (265.38 – 291.77)	561.44 (519.76 – 606.99)	116.84 (113.04 – 120.93)	125.31 (119.25 – 131.42)	67.51 (62.83 – 72.67)	124.15 (107.99 – 143.66)	59.03 (53.83 – 65.53)	183.59 (165.03 – 203.95)	25.55 (22.44 – 28.95)	95.12 (82.57 – 107.86)	8.54 (7.06 – 10.31)	32.75 (27.29 – 38.65)
Percentage change of incidence, 1992–2021 (%)	102.31 (87.12 – 118.72)		7.23 (5.51 – 8.62)		83.77 (71.94 – 97.63)		211.34 (206.49 – 211.98)		271.52 (367.71 – 372.63)		382.11 (387.39 – 374.92)	
Age-standardized incidence												
Rate per 100,000	23.14 (22.12 – 24.27)	27.51 (25.46 – 29.75)	46.98 (45.45 – 48.63)	42.42 (40.36 – 44.49)	25.58 (23.83 – 27.5)	32.37 (28.13 – 37.49)	15.58 (14.24 – 17.26)	26.93 (24.21 – 29.93)	11.13 (9.81 – 12.57)	20.25 (17.6 – 22.91)	9.54 (7.9 – 11.51)	15.07 (12.62 – 17.7)
Estimated annual change of incidence (% per year)	0.47 (0.41 – 0.52)		−0.43 (−0.54 – −0.31)		0.81 (0.71 – 0.9)		1.82 (1.73 – 1.91)		2.03 (1.97 – 2.1)		1.51 (1.36 – 1.66)	
APC model estimates												
Net drift of incidence (% per year)	1.15 (1.08 to 1.21)		0.02 (−0.09 to 0.14)		1.24 (1.14 to 1.35)		1.95 (1.82 to 2.08)		2.33 (2.24 to 2.42)		1.92 (1.8 to 2.04)	

Parentheses for GBD estimates denote 95% uncertainty intervals and parentheses for net drift denote 95% CIs

*APC* age period cohort; *GBD* global burden of diseases; *SDI* sociodemographic index; *WCBA* women of childbearing age

### Temporal trends in BC incidence in WCBA across different age groups

**Fig. 2:**
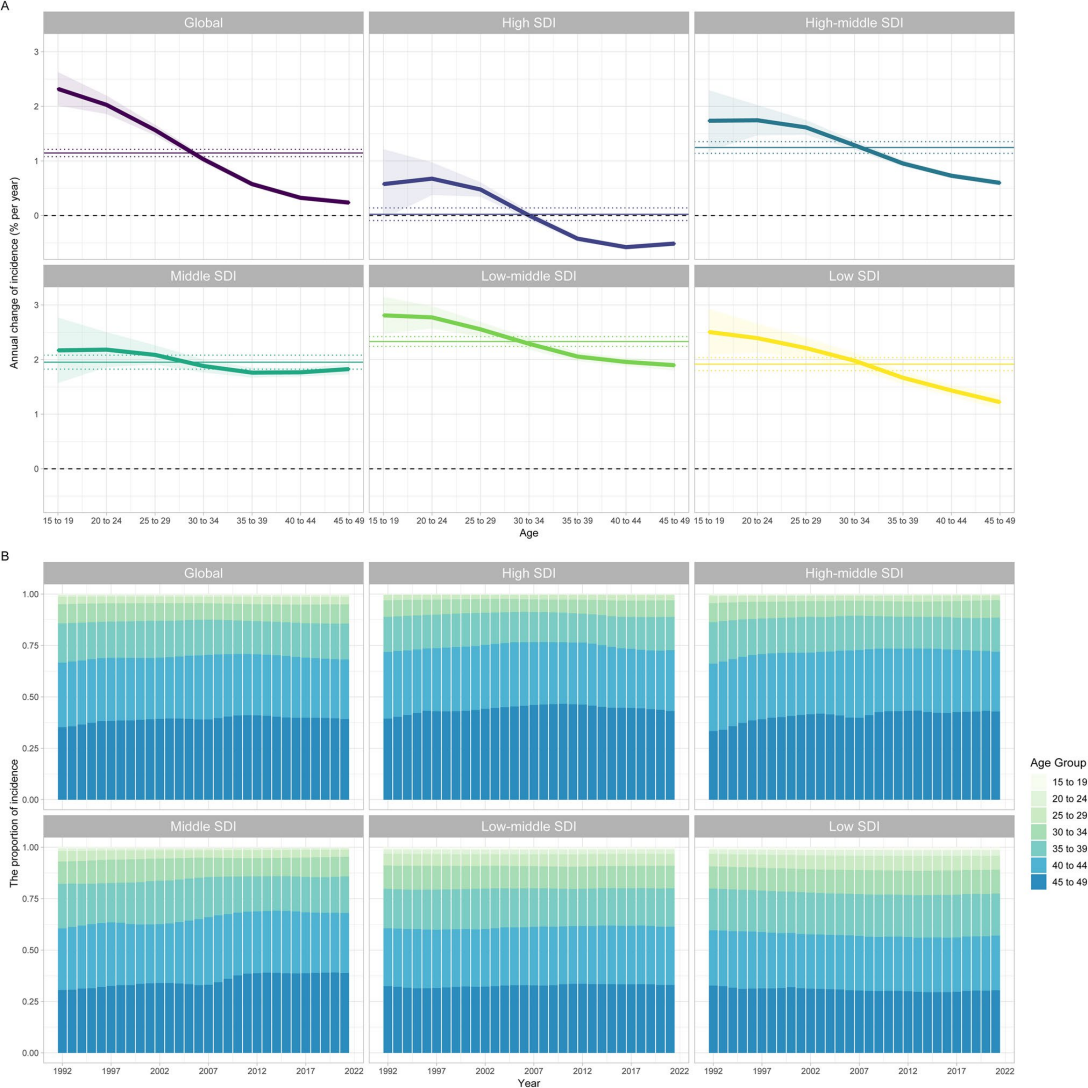
Local drift and age distribution of incidence from 1992 to 2021 for BC in WCBA across SDI quintiles. **A** Local drift of incidence rates for seven specified age groups of WCBA over the study period. **B** Temporal shifts in the age distribution of BC incidence among WCBA from 1992 to 2021. *BC* breast cancer; *SDI* sociodemographic index; *WCBA* women of childbearing age

Figure 2A and Table S2 illustrate the annual percentage changes in BC incidence across different age cohorts of WCBA, along with the local drift values derived from the APC model. Globally, BC incidence rates exhibit a clear upward trend from the youngest age group of 15–19 years to the oldest of 45–49 years. However, this trend moderates with age, peaking at 2.30% (95% CI 2.01–2.63%) during the adolescent years of 15–19, and tailing off to a gentler 0.24% increase (95% CI: 0.19–0.28%) in the mature adult age group of 45–49 years. Additionally, BC incidence among WCBA shows an increasing trend from the 15–19 to 25–29 years age group, stabilizes for the 30–34 years group, and then declines from the 35–39 to 45–49 years age group. Within these age groups, BC incidence rates rise in the High-middle, Middle, Low-middle, and Low SDI quintiles, but this upward trend lessens as age progresses. Table S3 presents the local drift of incidence rates for each specific location.

Figure 2B illustrates the temporal trends in BC incidence across various SDI quintiles. The data reveal a significant shift in incidence over time, with a transition from adolescence (ages 15–19) to middle and later adulthood (ages 20–49). This pattern is particularly pronounced in regions classified within the High-middle, Middle, and Low-middle SDI quintiles. Incidence in these regions is observed to increase consistently with age, especially after the age of 35, indicating heightened vulnerability within these age groups. Notably, the proportion of BC incidence in the older age group (45–49 years) has been steadily increasing, which may reflect an accumulation of risk factors or shifts in the epidemiological profile of the disease.

### Age, period, and birth cohort effects on BC incidence in WCBA

**Fig. 3:**
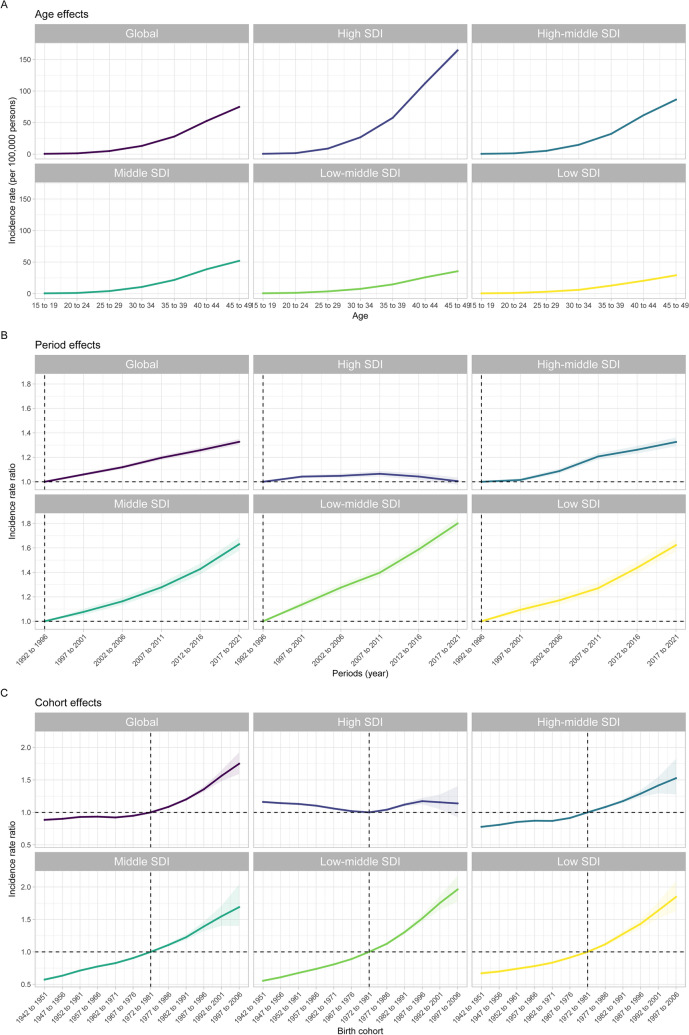
Age, period, and birth cohort effects on BC incidence in WCBA across SDI quintiles. **A** Age effects are depicted through the longitudinal age-specific rates for a set of birth cohorts, adjusted for period-specific deviations. **B** Period effects are shown as the relative risk of incidence (incidence rate ratio) between the periods 1992–1996 and 2017–2021, with the baseline period set at 1992–1996. **C** Birth cohort effects are represented by the relative risk of incidence (incidence rate ratio) comparing the cohorts born between 1942–1951 and 1997–2006, with the reference cohort being 1972–1981. The plotted points and shaded areas represent the incidence rates or rate ratios along with their 95% confidence intervals (CIs). *BC* breast cancer; *SDI* sociodemographic index; *WCBA* women of childbearing age

Figure 3 and Tables S4–S6 display the age, period, and birth cohort effects on BC incidence as derived from the APC model. A distinct age gradient characterizes BC incidence, with the youngest age group (15–19 years) recording the lowest rates and the oldest age group (45–49 years) the highest. Concurrently, the lowest SDI quintile consistently showed lower incidence across all age groups in comparison to higher SDI quintiles. Period effects indicated an increasing incidence risk across most SDI quintiles, except for the High SDI quintile, which exhibited an initial rise followed by a decline in risk. The High SDI quintile generally experienced lower period risks throughout the study period, in contrast to other quintiles, which faced higher risks for the majority of the time. Relative to the reference period of 1992–1996, the period risk for 2017–2021 varied from 1.01 (95% CI 0.98–1.03) in the High SDI quintile to 1.80 (95% CI 1.76–1.84) in the Low-middle SDI quintile. For birth cohort effects, there was an initial period of stability followed by an increasing risk of incidence in successive cohorts on a global scale. The High SDI quintile showed a pattern of initial improvement, followed by deterioration, and subsequent improvement, whereas incidence in other SDI quintiles showed a continuous decline. Compared to the reference cohort born between 1972–1981, the relative cohort risk for those born in the 1997–2006 cohort increased from 1.14 (95% CI 0.92–1.40) in the High SDI quintile to 1.96 (95% CI 1.77–2.17) in the Low-middle SDI quintile.

**Fig. 4:**
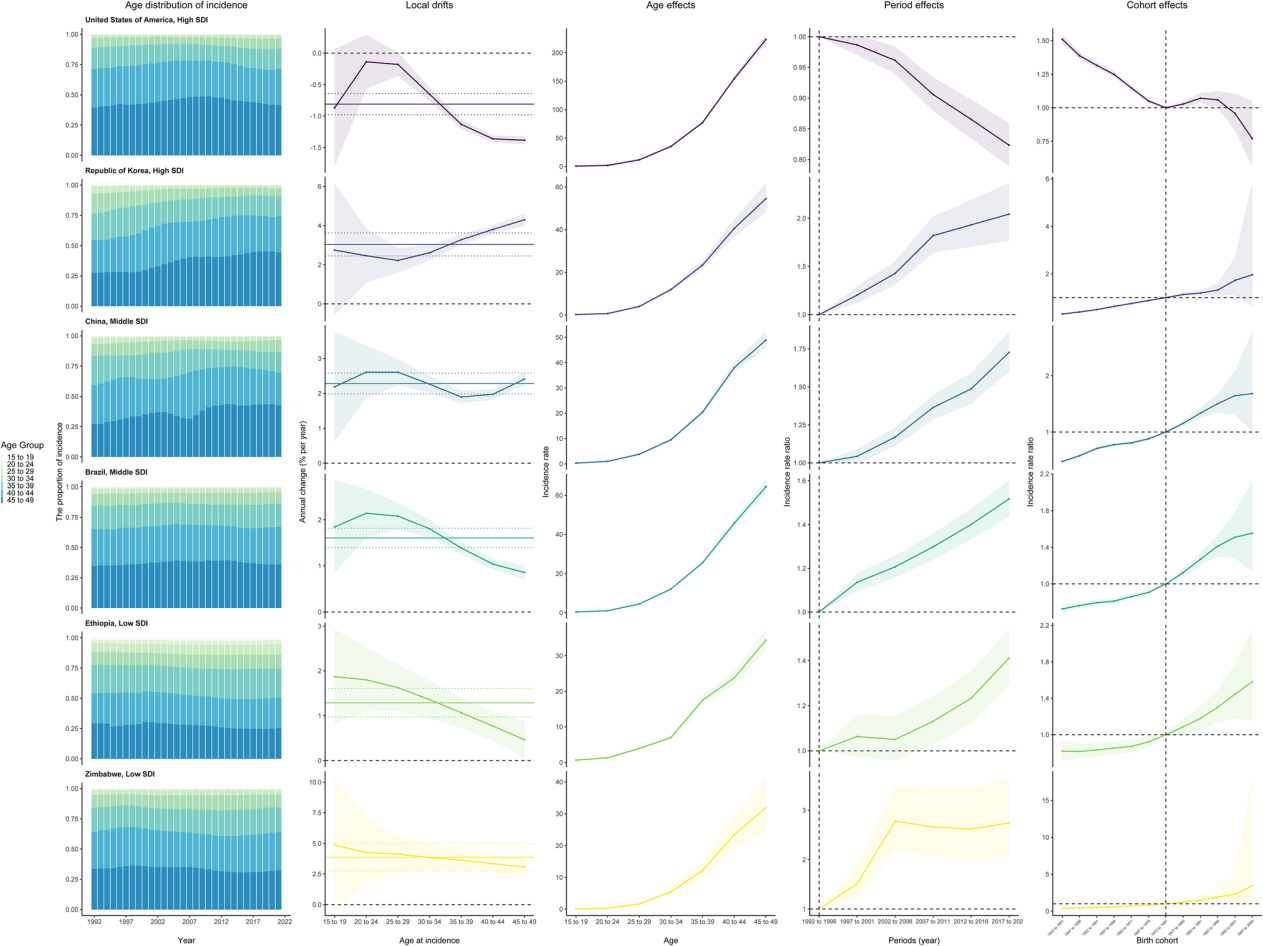
Age, period, and birth cohort effects on BC incidence in WCBA in exemplary countries. The age distribution of incidence illustrates the temporal shifts in the relative proportions of incidence across seven age groups ranging from 15 to 49 years, from 1992 to 2021. Local drift represents the annual percentage change in age-specific incidence rates for the same age groups, calculated from 1992 to 2021. *BC* breast cancer; *WCBA* women of childbearing age

Figure 4 captures the temporal trends in BC incidence for select countries across various SDI quintiles, highlighting both favorable and unfavorable patterns in age, period, and birth cohort effects. The USA, representing high SDI countries, exhibits highly favorable trends, with a local drift of less than 0% per year across all age groups, and a significant decline in risks over successive periods and birth cohorts. In contrast, Korea shows unfavorable trends, with no reduction in incidence across all age groups and a worsening of period and cohort risks in recent years. China and Brazil, representing the middle SDI quintile, illustrate an emerging shift in BC incidence from adolescence to adulthood. In both China and Brazil, older age groups are increasingly accounting for the growing proportion of BC incidence. Both period and cohort effects in these populous nations exhibit similar patterns, reflecting an escalating risk over the study period. Zimbabwe and Ethiopia, part of the low SDI quintile, display alarming trends in BC incidence. Despite rising global awareness and medical progress, these countries have not seen a decline in BC rates across all age groups; instead, they exhibit a net drift of 3.87 and 1.29, respectively. Furthermore, period and cohort effects are intensifying the risk, signaling ongoing deterioration in recent years.

## Discussion

Women diagnosed with BC face an elevated risk of severe pregnancy complications, potentially leading to significant maternal morbidity and mortality (Boere et al. 2022). To the best of our knowledge, this study pioneers the application of an APC model to examine temporal trends in BC incidence among WCBA worldwide and facilitates cross-country comparisons.

Between 1992 and 2021, BC incidence increased globally, with marked heterogeneity among SDI quintiles and age groups. The global female population expanded by 44.2%, and notably, the BC incidence among WCBA surged by 102.31%. The widespread pandemic of work and life stress could indirectly affect BC incidence by impacting the endocrine system (Brown et al. 2020). Moreover, increased health awareness and better access to health services have likely contributed to the rise in early diagnoses through screenings like mammography (Schünemann et al. 2020). This increase in detected cases subsequently results in the reported rise in BC incidence. The most significant rise in new BC cases among WCBA was noted in the Low SDI quintile, mainly attributed to population growth. Regions with higher SDI levels exhibit elevated age-standardized breast cancer incidence among WCBA, consistent with existing literature (Sun et al. 2021; Xu et al. 2023; Li et al. 2022). The highest disease prevalence was observed in countries with Low-middle and Middle SDI, particularly among women aged 35–49 years. These regions exhibited substantial annual percentage changes (APC) in BC incidence, reflecting the influence of both lifestyle transitions and healthcare disparities. On one hand, unhealthy lifestyles—encompassing high-fat diets and minimal physical activity—can heighten the risk of BC (Michaels et al. 2023). Additionally, urbanization and shifts in reproductive behaviors, such as delayed childbirth or having fewer children, also contribute (Shin et al. 2023; Victora et al. 2016). Conversely, in areas with higher SDI, WCBA may more frequently use hormonal contraceptives or undergo hormone replacement therapy, potentially elevating their BC risk (Dreyer et al. 2018). Nevertheless, the most pronounced increase in incidence rates was detected in the Low-middle and Middle SDI quintiles. Women in these regions are increasingly adopting lifestyles reminiscent of those in high SDI areas (Jahnke et al. 2022). Such shifts can significantly amplify the risk of BC. This trend mirrors the swift socioeconomic development and advancements in public health within Low to Middle SDI regions. However, it also underscores the necessity for increased vigilance and targeted interventions to mitigate BC risk factors in these areas.

Age effects demonstrate consistent patterns across various SDI regions, highlighting an increased risk with advancing age. Specifically, BC incidence, which is strongly age-related, is notably higher among the elderly (Heer et al. 2020). Furthermore, the risk of mortality also shows an increasing trend with age (Mubarik et al. 2022). Age-specific trends revealed that while BC incidence was lower among younger WCBA (15–29 years), the rate of increase in incidence was higher in this demographic compared to older WCBA (30–49 years). For instance, the 15–19 and 20–24 age groups showed local drift values exceeding 2.0% in Low and Low-middle SDI regions, highlighting the need for early interventions. Conversely, High SDI regions demonstrated a plateau or decline in incidence, particularly among women aged 45–49 years, likely due to the widespread adoption of preventive measures and early detection programs. Analyzing trends in BC incidence and mortality among Chinese women from 1990 to 2019 revealed age as a predominant influencing factor (Liu et al. 2022). Globally, the upward trend in BC incidence among WCBA is shifting towards younger ages, posing new challenges for healthcare system development and policy formulation. Breast cancer diagnosis and treatment can disrupt young women's career progression and reproductive decision-making, potentially impacting their fertility and straining family life and social relationships (Liu et al.^ 2021^). They also might not possess sufficient financial independence to cover the expenses associated with breast cancer treatment. Moreover, following a breast cancer diagnosis, young women may confront severe psychological challenges, including anxiety, depression, and diminished self-esteem (Dinapoli et al. 2021; Tsaras et al. 2018). Such issues can degrade their quality of life and potentially affect treatment outcomes and the recovery process. These challenges can exert a lasting adverse effect on various aspects of their subsequent life stages.

Globally, there is a noted increase in unfavorable period and cohort effects. The risk of BC incidence has escalated in recent decades, potentially due to a convergence of concerning cumulative risk factors. Unfavorable cohort effects were prominent in Low and Low-middle SDI countries, with successive birth cohorts showing heightened risks. This may be attributed to cumulative exposures to risk factors such as sedentary behavior, dietary shifts, and increasing use of hormonal contraceptives. In contrast, favorable cohort effects were observed in High SDI countries, particularly among women born after 1996, likely due to improved access to healthcare and earlier adoption of risk-reduction strategies. These factors encompass unhealthy lifestyles, heightened stress, and exposure to harmful by-products of industrialization and urbanization (Dong et al. 2021; Kadakia and Galea 2023). Evidence indicates a marked deterioration of these risk factors in recent years, underscoring an urgent need to bolster disease management and prevention strategies. Regarding cohort effects, individuals born more recently exhibit a higher overall risk compared to those born earlier. Later-born cohorts may delay childbirth or have fewer children, both factors linked to an elevated risk of breast cancer (Nichols et al. 2019; Barber et al. 2019). Furthermore, early-life exposure to social, behavioral, and environmental factors has been shown to influence BC outcomes (Michaels et al. 2023). Later-born individuals may also have been exposed to higher levels of endocrine-disrupting chemicals present in plastics, personal care products, and the environment, potentially elevating the risk of BC by disrupting hormonal balance (Braun 2017). Examining period and cohort effects allows us to discern the sources of incidence trends by period and birth cohort for each region, informing the efficacy of BC-related healthcare services. Across most SDI quintiles, the combined impact of period and cohort effects has contributed to the worsening of incidence rates. Moreover, significant heterogeneity exists in the influence of age, period, and birth cohort effects on BC incidence among WCBA across 204 countries and territories, revealing unique disease patterns that necessitate tailored health policy approaches at the national level.

The United Nations has set forth Sustainable Development Goal 3, which aims to reduce the global maternal mortality ratio. Enhancing early management of BC among WCBA significantly contributes to achieving this objective. Despite this, current research and policy focused on BC in WCBA remain limited. It is crucial to closely monitor the clinical status of these women, manage disease activity, and adjust medication regimens appropriately before and during pregnancy. One study delves into strategies for BC screening during pregnancy, underscoring the critical nature of early detection in high-risk scenarios for safeguarding maternal and child health (Lambertini et al. 2021). Another study reports on the use of letrozole to lower serum estradiol levels during superovulation, thereby minimizing hormone exposure for breast cancer patients before chemotherapy (Kasuga-Yamashita et al. 2022). This approach aids in preserving fertility while maintaining the success rates of in vitro fertilization treatments. There is a pressing need for increased research to deepen our understanding of the biological mechanisms behind BC in young women and to devise strategies that lessen the effects of cancer and its treatments on fertility and pregnancy outcomes. This study also underscores the necessity of tailoring public health interventions to specific age groups and SDI regions. For younger WCBA in Low and Low-middle SDI regions, targeted strategies such as awareness campaigns and resource allocation for screening programs could mitigate the rising incidence rates. Simultaneously, sustaining and expanding preventive measures in High SDI countries is essential to maintain declining trends.

Our study distinguishes itself from previous GBD publications by offering a more nuanced understanding of disease trends (Xu et al. 2023; Li et al. 2022, 2019; Mubarik et al. 2022), leveraging data to craft actionable public health insights. Specifically, by scrutinizing period and cohort effects, we can pinpoint the origins of incidence trends by period and birth cohort for each country, thereby informing the efficacy of BC healthcare services for WCBA. Additionally, estimating local drift values allows us to discern temporal incidence trends within each age group, with adjustments made for period effects. However, our study encounters several limitations. Firstly, cancer data might be underreported in low SDI regions due to insufficient diagnostic resources, which could inadvertently overstate the prevalence in regions with higher SDI. Secondly, the reliance on modeled data in GBD, particularly at the country level, stems from the GBD's extensive use of statistical modeling methods and the scarcity of raw data. This reliance could impact the precision of age, period, and birth cohort effect estimates. Thirdly, the GBD study does not account for ethnicities, races, or etiologies, which are potential determinants of BC incidence.

## Conclusion

In conclusion, our study provides a critical examination of the escalating incidence of BC among WCBA worldwide, uncovering notable disparities across SDI quintiles. The findings underscore an imperative for targeted public health interventions, strategies for early detection, and treatments that prioritize fertility preservation. Considering the intricate dynamics between lifestyle, environmental, and socio-economic factors, it is crucial to intensify research efforts, enhance diagnostic capabilities, and tailor healthcare solutions. These measures aim to lessen the impact on women affected by BC and to advance towards the United Nations Sustainable Development Goal 3.

## Supplementary Information

Below is the link to the electronic supplementary material.

Supplementary file1 (PDF 1107 KB)

## Data Availability

No datasets were generated or analysed during the current study.
